# Climbing Mountains: Building a Data Capture and Correction System for JUNGFRAU 9M

**DOI:** 10.1063/4.0000827

**Published:** 2025-10-27

**Authors:** Graeme Winter, Nick E Devenish, James O'Hea, Gary Yendell

**Affiliations:** 1 NE-CAT, Lemont, IL, USA; 2 Cornell University, Ithaca, NY, USA; 3 Diamond Light Source, Didcot, Oxfordshire, United Kingdom

## Abstract

The JUNGFRAU detector is a charge integrating, high-frame-rate, imaging detector developed by the PSI detector group, originally to support the SwissFEL Aramis beamline. The detector includes adaptive gain switching to give single photon sensitivity whilst also allowing sufficient analogue dynamic range to record ∼10,000 12keV photons. The use of adaptive gains requires a multi-stage data correction procedure, factoring in the pedestal and gain value for each pixel for each gain mode: at 2kHz frame rate this makes for a non-trivial undertaking.

At the SLS a data capture system for this has been developed, JUNGFRAUJOCH which uses a number of Xilinx FPGA boards to read the UDP data stream and perform the correction and the initial stage of data compression, before reading out to the CPU for compression. Whilst this is an effective solution, the technology choice of FPGA high level synthesis for programming has a very high barrier to entry. Some kind of hardware acceleration is however critical to ensure the correction and compression keep up with the data acquisition rate over the long term.

At Diamond we are also acquiring a JUNGFRAU 9M detector, to use for rotation and serial crystallography on beamline I24. This brings us to the challenge: adopt JUNGFRAUJOCH or develop an alternative system, since the wider view of high throughput computing has changed over the last few years. Here we present an alternative system build around the NVIDIA Grace Hopper Superchip (GH200) which includes a 72 ARM NEOVERSE v2 CPU cores, an NVIDIA H100 GPU and high bandwidth memory (Figure 1). The capabilities of this system allow us to consider building a data capture, correction and initial analysis system to support the JUNGFRAU 9M detector at Diamond Light Source, using far more mainstream technologies (CUDA for the accelerator programming, SLS detector for the front-end data capture) and offering the opportunity to tailor the system to fit in with the use cases being developed at Diamond Light Source beamline I24. This development also takes the opportunity to explore alternative methods for representing the data, keeping the data from modules separated allowing parallelism in data capture and analysis.

